# Effectiveness and Validity of Preoperative Ultrasonographic Airway Assessment and Clinical Screening Tests to Predict Difficult Laryngoscopy: A Prospective, Observational Study

**DOI:** 10.7759/cureus.41933

**Published:** 2023-07-15

**Authors:** Mamta Harjai, Sharif Alam, Shivani Rastogi, Sumit Kumar

**Affiliations:** 1 Anesthesia and Critical Care Medicine, Dr. Ram Manohar Lohia Institute of Medical Sciences, Lucknow, IND; 2 Anaesthesia, Hinchingbrooke Hospital, Huntingdon, GBR

**Keywords:** ultrasonography, mallampatti grading, laryngoscopy, cormack-lehane grade, airway

## Abstract

Background: The anticipation of a challenging airway can be demanding in emergency care settings. Due to the patient’s clinical condition, executing the pre-intubation clinical screening tests during the management of the airway in an emergency situation can be sometimes troublesome. Ultrasonographic airway assessment may become a helpful tool, but no specific sonographic measurements can precisely visualize the prospect of meeting a difficult airway. Therefore, the present study aimed to verdict some correlation between preoperative sonographic airway assessment parameters and the Cormack-Lehane (CL) grading at laryngoscopic view in patients undergoing general anesthesia with endotracheal intubation.

Methods: This observational study was conducted on 150 elective surgery subjects undergoing general anesthesia. The clinician in the pre-anesthetic clinic performed clinical airway and ultrasonographic airway assessments to predict difficult intubation and correlated with the CL grade viewed at laryngoscopy in the operative room during intubation. The parameters assessed were sensitivity, specificity, positive predictive value (PPV), and negative predictive value (NPV).

Results: In this study, the incidence of difficult intubation was 13.3%. The Mallampatti Grading (MPG) showed the maximum receiver operating characteristic (ROC) and area under the curve (AUC) among the clinical predictors, with 86.7% sensitivity. At the same time, the skin-to-hyoid distance has the maximum ROC among the sonographic parameters, and the skin-to-thyroid isthmus has the utmost sensitivity to predict difficult laryngoscopy.

Conclusions: Among the clinical predictors, MPG and the sonographic parameters, like the skin-to-hyoid distance and skin-to-thyroid isthmus, are favorable predictors of difficult laryngoscopy.

## Introduction

One of the prime responsibilities of the anesthesiologist is to provide adequate ventilation by securing the patient's airway. Due to the patient’s clinical condition, the pre-intubation clinical screening tests cannot always be executed, especially in emergency intubations. Poor handling of difficult airways increase morbidity and mortality of the patients. Preoperative appraisal of the patient’s airway facilitates the anesthesiologist to predict the difficulty of visualizing the glottis and whether intubation can be performed efficiently. However, there are numerous traditional clinical airway assessment parameters such as the history of snoring, obstructive sleep apnea, modified Mallampati grading (MPG) [1-2], inter-incisor gap (IIG), hyomental distance, thyromental distance (TMD), neck circumference (NC), upper lip bite test, body mass index (BMI), ability to flex and extend the cervical spine, etc. which are typically used to anticipate a difficult airway preoperatively [3-4]. Still, despite using these parameters, the diagnostic precision of a pre-anesthetic airway checkup in predicting difficult intubation is very low [5], and unpredicted difficult intubations persist. The diagnostic accuracy of these pre-anesthetic airway screening parameters has varied results in various studies [6].

Recently, ultrasound (U/S) in anesthesiology-related airway assessment and procedural interventions is showing encouraging results. Using U/S to assess the difficult airway constitutes another practical application of this versatile technology. U/S can estimate tracheal tube size, confirm correct tracheal tube and laryngeal mask placement, diagnose upper airway pathology, guide percutaneous tracheostomy or cricothyroidotomy, and predict post-extubation stridor [7]. However, few research studies have studied the role of ultrasonographic measurements in detecting difficult airways preoperatively. The objective and rationale behind our study were to search for a simple and bedside noninvasive method to identify difficult airways and determine whether the ultrasonographic assessment has any advantage over clinical screening tests in the evaluation of difficult airways preoperatively.

## Materials and methods

This prospective, comparative, observational, double-blind study was approved by the Institutional Ethical Committee (Dr. RMLIMS IEC no.77/17, CTRI/2019/02/017508), Dr Ram Manohar Lohia Institute of Medical Sciences, Lucknow, UP, and conducted over 12-15 months. The study included 150 patients, aged 18-65 years of either sex, with ASA grades 1 and 2, without any evident airway pathology posted for elective surgery under general anesthesia. The procedure was explained to all patients and informed consent was taken. Exclusion criteria included patients who denied consent or those with prominent features of the challenging airway with facial abnormalities, morbid obesity (body mass index, BMI >35 kg/m2), restricted mouth opening, full stomach patients necessitating rapid sequence intubation, and cervical spine anomalies, uncooperative and pregnant patients.

The anesthesiologist blinded to the study submitted all patients to an exhaustive preoperative airway assessment the day before surgery. The clinical parameters, like the modified MPG, IIG, TMD, neck circumference to thyromental distance ratio (NC/TMD), and BMI, were noted according to standard protocol and recorded for all patients.

After clinical screening tests, all patients went in detailed preoperative ultrasonographic evaluation by the anesthesiologist, who was competent and trained in airway U/S. Ultrasonographic airway evaluation was done with a linear high-frequency probe (L14‑5/38, frequency 14‑5 MHz) and the curvilinear low-frequency probe (C5‑2/60, frequency 7‑3 MHz) of the Sonosite max machine (Fujifilm Sonosite, Washington DC). U/S, the same person did guided measurements of all patients.

**Figure 1:**
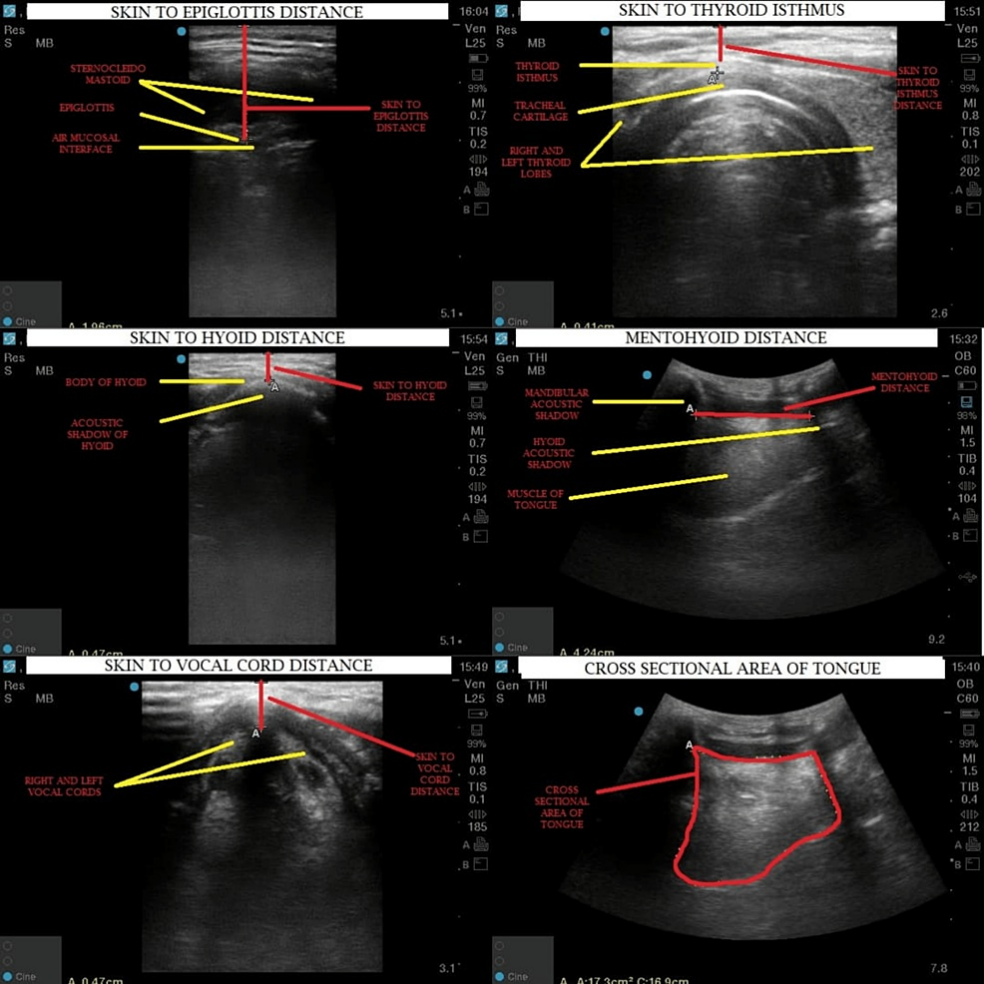
Various ultrasonographic parameters.

**Figure 2:**
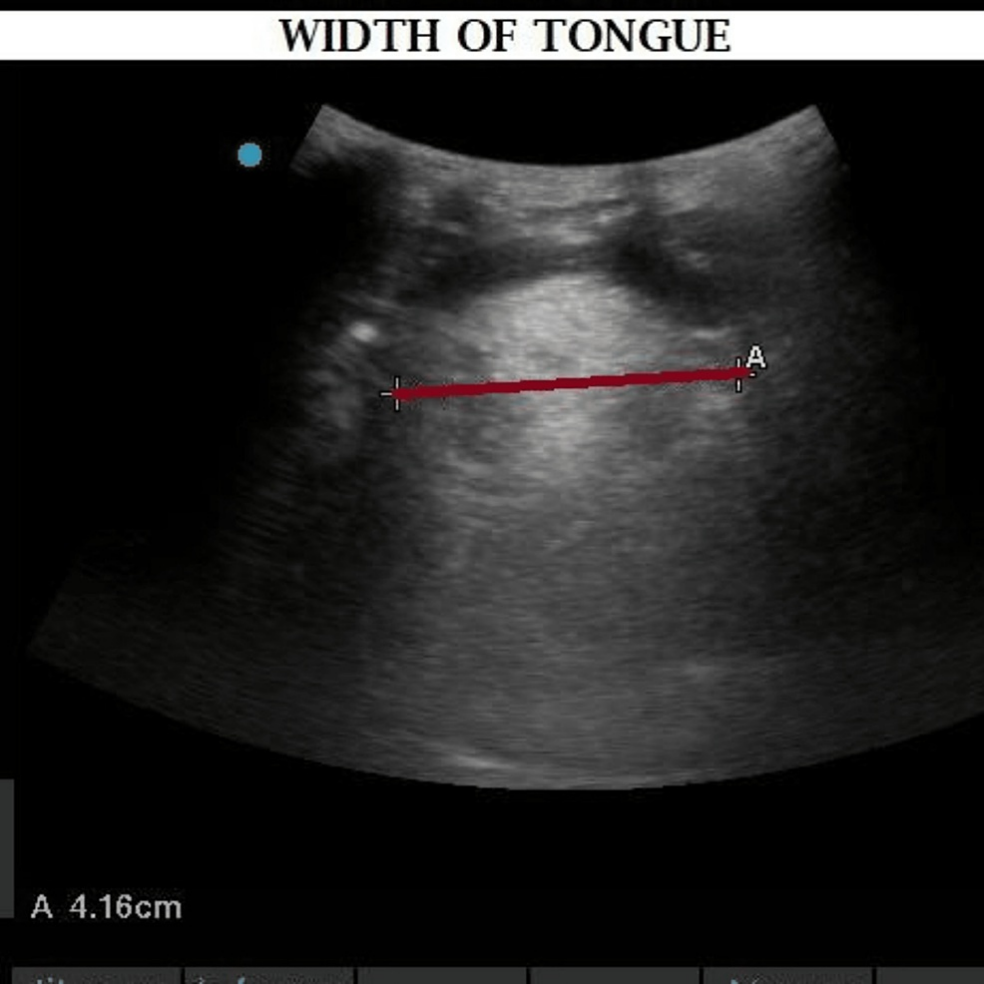
Width of tongue.

The sonographic measurement of the airway was achieved with the patient supine and the head and neck in the neutral position without a pillow, looking straight ahead with the mouth closed. Transverse views were used for measuring the width of the tongue, skin-to-hyoid distance, skin-to-epiglottis distance, skin-to-vocal cord distance, skin-to-thyroid isthmus distance, and the mid‑sagittal view for measuring the cross‑sectional area of the tongue and the mentohyoid distance as shown in Table 1. All measurements were recorded and noted (Figures 1-2).

**Table 1:** Parameters measured by U/S, the view and the type of probe used. U/S, ultrasound; USG, ultrasonogram

Parameter	View	Probe	Explanation
Width of the tongue	Transverse	Curvilinear	The most distant points on its upper surface were by transverse scan at the midsection of the tongue
Skin-to-hyoid distance (measured from skin to anterior surface of hyoid bone)	Transverse	Linear	Hyoid bone visible as a superficial hyperechoic inverted U-shaped linear structure
Skin-to-epiglottis distance (measured from skin to anterior surface of epiglottis at the level of thyrohyoid membrane)	Transverse	Linear	Epiglottis is visible as a hypoechoic curvilinear structure between the hyoid and the thyroid
Skin-to-vocal cord distance (measured from skin to anterior commissure of vocal cords)	Transverse	Linear	Two hypoechoic structures form an isosceles triangle with a central tracheal hollow shadow
Skin to thyroid isthmus distance (measured from skin to anterior surface of tracheal cartilage at the level of thyroid isthmus)	Transverse	Linear	The homogenous hyperechoic bilobed structure is connected with the isthmus as the linear probe moves caudally from the thyroid notch
The cross-sectional area of the tongue	Midsagittal	Curvilinear	It is measured between the two hyperechoic structures, the mentum and hyoid bone, using the calculation option provided in USG
Mentohyoid distance	Midsagittal	Curvilinear	Distance between two hyperechoic structures, cranially the mentum and caudally the hyoid, using the caliper option in the ultrasound machine

After a preoperative assessment, the patient was given IV access in the operating room. All standard monitors like electrocardiogram (ECG), non-invasive blood pressure (NIBP), pulse oximetry, and temperature probe were connected, and values were noted. After 3-5 min of preoxygenation, i.v. midazolam (0.05-0.15 mg/kg) and fentanyl (2μg/kg) were administered as premedication. Next, the patients were induced by an i.v. induction agent (Propofol 2 mg/kg). After administration of i.v. muscle relaxant, patients were intubated by a senior anesthesiologist who had the experience of a minimum of 10 years and was blinded to the findings of preoperative airway evaluation. Direct laryngoscopy was conducted using a Macintosh blade (Intersurgical, Berkshire, UK), and Cormack-Lehane’s (CL) grade was observed and noted. Intubation was classified as easy (CL Grade 1 and Grade 2) or difficult (CL Grade 3 and Grade 4).

Statistical analysis

Statistical analysis was done using commercially available computer software IBM SPSS software version 21 for Windows IBM Corp., Armonk, NY). The results were averaged (mean ± standard deviation) for each parameter for continuous data. The sensitivity, specificity, positive predictive value (PPV), and negative predictive value (NPV) were calculated for all the measured clinical and ultrasonographic parameters. The correlation between different predictors and difficult laryngoscopy was evaluated using binary univariate and multivariate logistic regression. Pearson's correlation coefficient assessed the correlation between significant ultrasonographic and clinical predictors. A p-value <0.05 was interpreted as a significant value. We did a receiver operating characteristic (ROC) analysis to determine the discriminative power of individual tests and the combination. The area under the curve (AUC) with a 95% confidence interval was calculated. The ROC curve graphically displays sensitivity and specificity, and the AUC measures the inherent validity of the test. The maximum AUC value of 1 indicates a perfect diagnostic test.

## Results

The sample size of 150 adult patients included 64 men (42.7%) and 86 women (57.3%), with ages ranging from 18 to 65 years. The age, gender, height, and BMI demographic profile were comparable in the easy and challenging laryngoscopy group (Table 2).

**Table 2:** Demographic data of the easy and challenging laryngoscopy groups. p = binary univariate logistic regression *Significant (p = <0.05)

Parameters	Easy (CL 1-2) (n=130)		Difficult (CL 3-4) (n=20)		p-value
	Mean	±SD	Mean	±SD	
Age (years)	43.75	12.24	47.90	10.61	0.154
M:F (sex)	57 : 73	43.85% : 56.15%	7 : 13	35% : 65%	0.720
Weight (kg)	61.46	11.31	67.30	7.67	0.027^*^
Height (cm)	158.72	8.10	157.65	10.38	0.599
BMI (kg/m2)	24.31	3.80	27.015	4.72	0.005^*^

The incidence of difficult laryngoscopy was 13.3% (20 patients), and the incidence of easy laryngoscopy was 86.7 % (130 patients). The distribution of the patients according to the CL grade at direct laryngoscopy showed CL Grade I (51.3%) in 77 patients and 53 patients with CL Grade II (35.3%). In comparison, 20 patients showed CL Grade III (13.3%), and none had CL Grade IV.

Clinical airway predictors like MPG, IIG, and various sonographic parameters like the width of the tongue, skin-to-hyoid distance, etc. were calculated to predict laryngoscopy depending on Cormack-Lehane’s (CL's) view as shown in Table 3. 

**Table 3:** Clinical and ultrasonographic parameters of the easy and difficult laryngoscopy groups. p = binary univariate logistic regression *Significant (p = <0.05) IIG, inter-incisor gap

Parameters	Easy (CL 1-2) (n=130)		Difficult (CL- 3-4) (n=20)		p-value
	N	%	N	%	
MPG score 1 2 3 4	37 76 17 0	28.46 58.46 13.08 0.00	1 12 4 3	5 60 20 15	<0.001^*^
	Mean (cm)	±SD	Mean (cm)	±SD	
IIG	4.80	0.65	4.08	0.90	<0.001^*^
Width of tongue	4.54	0.53	4.36	0.49	0.174
Skin-to-hyoid distance	0.59	0.22	0.73	0.21	0.009^*^
Skin-to-epiglottis distance	1.56	0.28	1.58	0.31	0.756
Skin-to-vocal cord distance	0.66	0.17	0.66	0.25	0.937
Skin to thyroid isthmus distance	0.37	0.15	0.45	0.14	0.021^*^
The cross-sectional area of the tongue	21.74 (cm2)	3.95	22.41 (cm2)	2.61	0.468
Mentohyoid distance	4.98	0.63	4.78	0.40	0.166

The MPG Grade III and Grade IV strongly predicted anticipated difficult airways. In addition, we found that the IIG was statistically significant between patients of the easy and challenging laryngoscopy group. Furthermore, U/S-guided airway assessment showed a statistically significant difference in the skin-to-hyoid distance and skin-to-thyroid isthmus distance of patients with easy and difficult laryngoscopy.

The binary multivariate logistic regression was used to analyze relevant clinical and ultrasonographic parameters to look for the potential independent risk factors for difficult laryngoscopy, as shown in Table 4. Among clinical parameters, the IIG, TMD, NC, and NC/TMD were significant independent risk factors for laryngoscopy. In contrast, skin-to-epiglottis was an important independent risk factor for laryngoscopy among ultrasonographic parameters.

**Table 4:** Binary multivariate logistic regression (forward-Wald) analysis to determine the independent risk factors for difficult laryngoscopy in each group. *Significant (p = <0.05) SE, standard error; IIG, inter-incisor gap; CI, confidence interval; OR, odds ratio

Variables	Beta	SE	Wald	p-value	OR	95% CI	
						Lower	Upper
Mallampati grade	0.755	0.783	0.931	0.335	2.128	0.459	9.866
IIG (cm)	-2.317	0.819	7.995	0.005^*^	0.099	0.020	0.491
Thyrometal distance (cm)	-18.266	6.487	7.928	0.005^*^	-	-	0.004
Neck circumference (cm)	4.737	1.666	8.089	0.004^*^	114.139	4.361	2987.00
NC/TMD	-35.90	13.219	7.378	0.007^*^	-	-	-
Width of tongue (cm)	-1.660	1.019	2.655	0.103	0.190	0.026	1.401
Skin-to-hyoid distance (cm)	-1.798	3.035	.351	0.554	0.166	0.000	63.454
Skin-to-epiglottis distance (cm)	-6.169	2.293	7.240	0.007^*^	0.002	0.000	0.187
Skin to vocal cord distance (cm)	3.205	2.091	2.349	0.125	24.646	0.409	1484
Skin to thyroid isthmus distance (cm)	5.466	3.723	2.156	0.142	236.613	0.160	348900
The cross-sectional area of the tongue (cm2)	-0.084	0.146	0.335	0.563	0.919	0.691	1.223
Mentohyoid distance (cm)	0.099	0.459	0.046	0.830	1.104	0.449	2.711

Based on the ROC curve, the AUC of clinical predictors like MG, IIG, NC/TMD was assessed along with ultrasonographic predictors like the width of the tongue, skin-to-hyoid distance, skin-to-epiglottis distance, skin-to-vocal cord distance, skin-to-thyroid isthmus distance as shown in Table 5. The AUC for modified MG among the clinical predictors and skin-to-hyoid distance among the ultrasonic predictors were 0.690 and 0.692, respectively, suggesting the maximum validity among the parameters studied.

**Table 5:** ROC analysis of difficult laryngoscopy components. ROC, receiver operating characteristic; SE, standard error; CI, confidence interval; IIG, inter-incisor gap

	Area	SE	p-Value	95% CI	
				Lower bound	Upper bound
Mallampati grade	0.690	0.062	0.006	0.568	0.812
IIG (cm)	0.268	0.061	0.001	0.149	0.388
NC/TMD	0.679	0.054	0.010	0.573	0.785
Width of tongue (cm)	0.391	0.067	0.116	0.260	0.521
Skin-to-hyoid distance (cm)	0.692	0.061	0.006	0.571	0.812
Skin-to-epiglottis distance (cm)	0.503	0.071	0.960	0.364	0.642
Skin to vocal cord distance (cm)	0.517	0.080	0.808	0.361	0.673
Skin to thyroid isthmus distance (cm)	0.579	0.055	0.258	0.471	0.686
The cross-sectional area of the tongue (cm^2^)	0.667	0.064	0.017	0.541	0.792
Mentohyoid distance (cm)	0.370	0.061	0.061	0.250	0.489

The sensitivity, specificity, NPV, and PPV of various clinical and ultrasonographic predictors were calculated to exhibit the validity of risk factors, as shown in Table 6. In the present study, the cut-off points for various tests to assess the difficult laryngoscopy were Mallampati score ≥3, IIG <3.8, skin-to-hyoid distance <0.785, skin-to-epiglottis distance <2.3, skin-to-vocal cord distance <0.785, skin-to-thyroid isthmus distance <0.25, the cross-sectional area of tongue <23, and mentohyoid length <5.26. Amidst all clinical variables, we found IIG to be the most sensitive (sensitivity of 89.3) and most specific (specificity of 50%) in predicting difficult laryngoscopy preoperatively. Mallampati score showed PPV and NPV as 100%, while IIG had a PPV of 96.2% and an NPV of 25%. We found skin-to-thyroid isthmus distance (88.5%) and skin-to-epiglottis distance (100%) among ultrasonographic parameters with maximum sensitivity and specificity.

**Table 6:** Sensitivity, specificity, PPV, and NPV of the conventional and ultrasonographic parameters in predicting difficult laryngoscopy. PPV, positive predictive value; NPV, negative predictive value; IIG, inter-incisor gap

Test	Sensitivity (%)	Specificity (%)	PPV (%)	NPV (%)
Mallampati score	86.7	13.3	100.0	100.0
IIG (cm)	89.3	50.0	96.2	25.0
Skin-to-hyoid distance (cm)	88.3	20.0	81.5	30.0
Skin-to-epiglottis distance (cm)	87.2	100.0	100.0	5.0
Skin to vocal cord distance (cm)	88.4	20.7	82.3	30.0
Skin to thyroid isthmus distance (cm)	88.5	8.7	83.8	10.0
The cross-sectional area of the tongue (cm^2^)	88.0	16.0	67.7	40.0
Mentohyoid distance (cm)	83.0	4.5	67.7	10.0

## Discussion

Several noninvasive and traditional screening parameters, like mouth opening, modified Mallampati classification, jaw protrusion, thyromental distance, and the upper lip bite test, are available for airway assessment during the pre-anesthetic examination. However, even with this, much work has been done on these parameters; their authenticity in predicting direct laryngoscopy while tested alone or in combination is questionable due to their low accuracy [8].

Therefore, a noninvasive bedside screening test should be emphasized to envisage the difficult airway more precisely during the pre-anesthetic examination. In the last few years, the inclusion of U/S in anesthesiologist's arsenal has revolutionized perioperative care, including pre-anesthetic assessment; however, few studies have employed ultrasonography-directed predictors to measure the airway during the preoperative period and shown encouraging results [9-10]. Therefore, the present study was intended to analyze preoperative sonographically assessed and traditional clinical parameters to predict the grade of difficulty at direct laryngoscopy.

In the present study, among clinical predictors for complex airway evaluation, the weight of patients and thus BMI (kg/m2) had predictability towards anticipation of the difficult airway (p = 0.005). Various researchers like Gonzalez et al. [11] and Brodsky et al. [12] found an increased incidence of difficult tracheal intubation in obese than in lean patients (14.3% vs. 3%; p = 0.03) and might be caused by fat deposition specifically in the anterior region of the neck in patients with high BMI.

Our study found MPG grades (III & IV) to be highly statistically significant (p < 0.001) for the detection of difficult intubation. The Mallampati test had a sensitivity of 86.7% in the present study. In comparison, it has shown a broad spectrum of sensitivity from 40% to 82.4% in various studies [2, 13-14], which could be ascribed to the inter-observer variability during the assessment. Therefore, the same investigator conducted the tests to minimize inter-observer variability in the present study.

 Among other clinical predictors, IIG was an independent risk factor (p < 0.001) for difficult laryngoscopy in both groups. IIG had a maximum sensitivity of 89.3% with 50% specificity though PPV and NPV were lower than MPG. Our results were akin to the studies by Abdel Raouf et al. [15] and Wilson et al. [16], who found laryngoscopy challenging in patients with a significantly smaller IIG.

The present study did not find mentohyoid distance, TMD, and NC significant. Shiga et al. also found the diagnostic value of TMD unsatisfactory as an individual predictor [6]. However, many previous studies [17-18] concluded that an increase in neck circumference significantly affected difficult intubation and was directly proportional to difficult intubation, especially in obese patients. And since we excluded the patient with BMI > 35 kg/m2 in the present study, this disparity in results is expected. But during multivariate binary logistic regression analysis to determine the independent risk factors, both NC (p = 0.004) and NC/TMD (p = 0.007) were found statistically significant in differentiating between easy and difficult laryngoscopy groups. The above finding was corroborated by many studies [19-21], and NC/TMD was concluded to be a compelling predictor in their conclusions. Another clinical predictor, ratio of height to thyro mental distance (RHTMD) (cut off -22.67), was also found statistically non-significant (p = 0.253) in contrast to the studies like Schmitt et al. [22] (cut-off of RHTMD > 25) and Krobbuaban et al. [23] (cut-off of RHTMD > 23.5) where it was concluded to be a good predictor for difficult laryngoscopy.

 Regarding parameters assessed by U/S in our study, we did not get the difference in tongue width in the easy and challenging laryngoscopy group as statistically significant (p = 0.174). The result was supported by Andruszkiewicz et al. [24], who found tongue width as a non-significant ultrasonographic predictor (p = 0.484).

 The present study, skin-to-hyoid distance, was statistically significant (p = 0.009) in easy and difficult laryngoscopy patients (Figure 4). This result was akin to many studies like Adhikari et al. [25] and Ji et al. [26], who found that ultrasonographic measurements of anterior neck soft tissue at the level of the hyoid bone were more significant in the difficult laryngoscopy group (p < 0.0001). Ezri et al. [18] also concluded that patients with more distance between skin and hyoid bone had difficult laryngoscopy. Kanoujiya et al. [27] conducted a similar study, in which they found the space between the skin and hyoid bone to be statistically significant, with a cut-off value of 0.81 cm, which is close to our result of statistical significance and cut-off deal of 0.785 cm for prediction of difficult laryngoscopy with sensitivity 88.3% and PPV 81.5%. So, skin-to-hyoid distance measured ultrasonographically could better anticipate the presence of a difficult airway.

 Another parameter, skin-to-epiglottis distance showed maximum specificity (100%) in our study with comparable sensitivity (87.4%) and was not statistically significant (p = 0.756) using binary univariate logistic regression. But multivariate logistic regression analysis found it to be a significant predictor of difficult laryngoscopy (p = 0.007). On the other hand, Petrișor et al. [28] did not get the skin-to-epiglottis distance as a significant predictor (p=0.60).

When measured ultrasonographically, the skin-to-vocal cord distance did not show any statistical significance and, therefore, was not considered a useful predictor in the present study. However, previous studies like Adhikari et al. [25] and Komatsu et al. [29] supported this finding.

 We also found that the skin-to-thyroid isthmus distance was statistically significant (p = 0.021) in our sample population by using binary univariate logistic regression although a study conducted by Ezri et al. [18] had conflicting results and did not find the skin-to-thyroid isthmus distance as a reliable tool. This discrepancy in outcome might be due to differences in the study population. They included morbidly obese patients in their study, and we excluded the patient with BMI >35 kg/m2. Amongst all parameters assessed by U/S in the present study, skin-to-thyroid isthmus had maximum sensitivity (88.5%) higher than skin-to-hyoid and skin-to-vocal cord distance. PPV (83.8%) was comparable but with low specificity (8.7%).

In the present study, the tongue's cross-sectional area did not significantly predict difficult intubation, and the findings concord with Wojtczak [10]. They did not find tongue-related ultrasonographic parameters statistically significant for difficult intubation; however, Andruszkiewicz et al. [24] found that larger tongue cross-sectional area and volume were associated with difficult laryngoscopy. Similarly, we did not find the mean mentohyoid distance measured by ultrasonography to be statistically significant (p = 0.166) in both groups, and this finding was corroborated by Petrișor et al. [28], who also did not suggest mentohyoid distance as a significant predictor for difficult laryngoscopy (p = 0.31).

The ROC curve was obtained for various clinical and ultrasonographic variables [30]. Diagnostic accuracy is precisely associated with the AUC. Our study found that MG was more considerable under the curve (AUC=0.690). At the same time, skin-to-hyoid distance (AUC = 0.692) has the maximum AUC among ultrasonogram (USG) parameters.

We encountered some limitations of the present study. It included 150 patients, and the incidence of difficult intubation was 13.3%, with none of the patients with CL Grade 4. Therefore, the sample size of our study may be limited. In addition, patients with BMI >30 kg/m2 were not included making it one potential study limitation. Another constraint of the present study is that U/S-guided airway evaluation requires the new emerging technique to have the exact values of sonographically measured cut-offs to determine difficult and normal airways. Hence, more contemporary studies with larger sample sizes and the inclusion of diverse groups are warranted to establish variables that can correlate more accurately with a difficult airway.

## Conclusions

The U/S-guided measurement of anterior soft tissue neck thickness at the level of hyoid and thyroid isthmus level can be potential diagnostic predictors for assessing difficult intubations in the preoperative period. A value of 0.785 cm and 0.25 cm for skin-to-thyroid isthmus distance is more sensitive than clinical parameters such as MPG in predicting a CL Grade of 3 or 4. We observed skin-to-epiglottis distance as moderately sensitive, with the highest specificity and maximum predictive value. In clinical screening tests, BMI, MPG, and IIG were significant predictors in differentiating easy and difficult laryngoscopy. In the future, ultrasonography-assisted parameters can offer substantial usage as a valuable noninvasive adjuvant in anticipating challenging airways along with clinical predictors in the preoperative phase.
